# Characterization of a xylanase-producing *Cellvibrio mixtus* strain J3-8 and its genome analysis

**DOI:** 10.1038/srep10521

**Published:** 2015-05-21

**Authors:** Yi-Rui Wu, Jianzhong He

**Affiliations:** 1 Department of Civil and Environmental Engineering National University of Singapore, 117576 Singapore

## Abstract

*Cellvibrio mixtus* strain J3-8 is a gram-negative, xylanase-producing aerobic soil bacterium isolated from giant snails in Singapore. It is able to produce up to 10.1 U ml^−1^ of xylanase, which is comparable to xylanase production from known bacterial and fungal strains. Genome sequence analysis of strain J3-8 reveals that the assembled draft genome contains 5,171,890 bp with a G + C content of 46.66%, while open reading frame (ORF) annotations indicate a high density of genes encoding glycoside hydrolase (GH) families involved in (hemi)cellulose hydrolysis. On the basis of 15 identified putative xylanolytic genes, one metabolic pathway in strain J3-8 is constructed for utilization of xylan. In addition, a 1,083 bp xylanase gene from strain J3-8 represents a new member of GH11 family. This gene is verified to be novel via phylogenetic analysis. To utilize this novel gene for hydrolysis of xylan to xylose, it is expressed in recombinant *E. coli* and characterized for its hydrolytic activity. This study shows that strain J3-8 is a potential candidate for hydrolysis of lignocellulosic materials.

Lignocellulosic materials represent the most abundant reservoir of organic carbon in the biosphere^1^. However, to use lignocellulosic materials as feedstock for fermentation, costly and environmental unfriendly acid or heat pretreatment are often required. To address this issue, recent studies have focused more on enzymatic hydrolysis of such materials into fermentable monomer sugars (e.g., glucose, xylose), which can be further converted into value-added products, such as biofuels^2^,^3^. In nature, some bacterial and fungal enzymes, such as cellulase and hemicellulase, are responsible for the hydrolysis of lignocellulosic materials to glucose and xylose^4^,^5^. With growing demand for utilization of lignocellulosic materials as biofuel feedstocks, searching for novel enzymes which exhibit unique characteristics is more important than ever before.

Bacteria from genus *Cellvibrio* are usually aerobic, gram-negative and cellulolytic. Most of them have been reported as degraders of cellulose, dextran, xylan, chitin and starch^2^,^6^. In particular, *Cellvibrio japonicus* has a powerful hydrolytic enzyme system, which permits the degradation of plant cell wall. Since hydrolytic enzymes expressed by *C. japonicus* do not assemble into large multienzyme cellulosome-like complexes, they can be excreted into culture media more easily^2^,^7^. Even though numerous hydrolytic enzymes and functional genes have been discovered, their biochemical properties remain largely unknown^2^. Recently, other species such as *Cellvibrio mixtus* was also found to be capable of hydrolyzing hemicellulose and cellulose^6^,^8^,^9^. Fontes *et al.* also demonstrated that *C. mixtus* can express internal xylanase when cultivating in a medium with xylan as a sole carbon source^6^. Some functional genes from this species involved in encoding xylanases or cellulases have also been identified and characterized^6^,^10^. On the other hand, the lack of genomic information hinders further understanding of *C. mixtus*’s glycoside hydrolases (GHs) system with complex architecture comprising of both catalytic modules and non-catalytic carbohydrate-binding modules (CBMs), while CBMs is crucial in enhancing the capability of GHs as well as improving their efficiency during the hydrolytic process^11^.

In order to provide comprehensive information on xylanase-producing *Cellvibrio* species, here we investigate xylanase production by a novel xylanolytic *C. mixtus* strain J3-8, report its draft genome sequence along with annotation on its xylanolytic enzyme, and reconstruct the main metabolic pathway involved in xylan utilization. Additionally, recombinant expression and biochemical characterization of a new xylanase (belonging to the GH11 family) in strain J3-8 was conducted in *E. coli* cells so as to distinguish it from other reported xylanases.

## Materials and Methods

### Isolation, identification and cultivation of xylanase-producing bacteria

Giant snails on the grassland in Singapore were collected as a source to screen for xylanase-producing bacteria. At 30 °C and a pH of 7.0, bacterial community was aerobically enriched by using mineral salts medium with xylan (5 g L^−1^) as the sole carbon source. After several transfers, colonies on agar plates were selected when showing xylanolytic activity as indicated by Congo red staining method. One colony with superior xylan degradation capability, named J3-8, was ultimately selected for the following investigation and phylogenetic identification based on 16S rRNA gene sequence. The phylogenetic analysis were performed by using program ClustalX (Version1.8.1) with alignment of multiple 16S rRNA gene sequences from different microbial species. The phylogenetic tree was established with neighbor-joining method by using program MEGA (Version 5.05) with distances determined according to Kimura’s two-parameter model and bootstrap values (>50%) based on 1,000 replicates.

Unless stated otherwise, the strain was aerobically grown in mineral salts medium with xylan (10 g L^−1^) as the sole carbon source and at its optimal conditions (30 °C, pH 7.0). The mineral salts medium contained: (g L^−1^) NaCl 1.0, MgCl_2_·H_2_O 0.5, KH_2_PO_4_ 0.2, NH_4_Cl 0.3, KCl 0.3, CaCl_2_·H_2_O 0.015; (mg L^−1^) FeCl_2_·4H_2_O 1.5, CoCl_2_·6H_2_O 0.19, MnCl_2_·4H_2_O 0.1, ZnCl_2_ 0.07, Na_2_MoO_4_·2H_2_O 0.036, NiCl_2_·6H_2_O 0.024, Na_2_WO_4_·2H_2_O 0.008, Na_2_SeO_3_·5H_2_O 0.006, H_3_BO_3_ 0.006, CuCl_2_·2H_2_O 0.002, 10 mM TES as buffer, and 0.5% of yeast extract as nitrogen source.

### Optimization of xylanase production from *C. mixtus* strain J3-8

Strain J3-8 was firstly activated overnight with mineral salts medium containing xylan and inoculated into the same medium for following on experiments. Optimization of xylanase production of strain J3-8 was conducted at various culturing conditions, including temperature (25–35 °C), pH (6.0–8.0), xylan concentration (5–10 g L^−1^), nitrogen source (addition of yeast extract, peptone or ammonium sulfate), and the inocula size (1–10%). After 5 days of incubation, the supernatant from culture medium was collected by centrifugation at 14,000 rpm and 4 °C for 10 minutes, which were used for xylanase activity analysis.

### DNA extraction, genome sequencing, ORF prediction and annotation

Cells of *C. mixtus* strain J3–8 were collected from 10 mL culture by centrifugation at 10,000 g for 10 min, then the pellet was washed by sterilized TE buffer twice to remove the residual medium before DNA extraction. The genomic DNA of *C. mixtus* J3-8 was extracted by using a Qiagen genomic DNA kit with genomic-tip process (Qiagen, Germany), and verified to be high quality (DNA amount: ≥20 μg and purity: 1.8 ≤ OD_260 nm/280 nm_ ≤ 2.0).

The genomic DNA was firstly sheared randomly into fragments by Covaris S/E210 bioruptor for DNA fragment library preparation. After the desired fragments were received, a 500-bp paired-end library was constructed for sequencing using high-throughput Illumina sequencing technology with an Illumina HiSeq 2000 sequencer (Illumina Inc.). The paired-end reads were assembled by using SOAPdenovo (version 1.05), and assembly errors were corrected by using SOAPaligner (version 2.21). After obtaining the draft genome sequence, open reading frames (ORFs) were identified by using Glimmer (version 3.02), and the putative protein coding sequences (CDSs) were functionally annotated by a series of reference databases, including GenBank, UniProtKB/TrEMBL, KEGG (Kyoto Encyclopedia of Genes and Genomes), COG (Clusters of Orthologous Groups) and UniProtKB/Swiss-Prot databases (identity threshold >40%). Genes for tRNA and rRNA were identified by tRNAscan-SE (Version 1.21) and rRNAmmer (Version 1.2), respectively. Searches for the xylan and cellulose related glycoside hydrolases (GHs) as well as carbohydrate-binding modules (CBMs) were performed based on the BLASTP and the CAZy nomenclature^12^. Putative signal peptides were predicted using the SignalP 4.0 server program^13^.

### Cloning and expression of a GH11 xylanase from *C. mixtus* strain J3-8 in *E.coli*

Among those annotated xylan hydrolyzing genes, one of the GH11 family genes (Tag No. CM1139) - encoding xylanase was amplified by using the designed primers Xyl-F (5’- GGCCCAAGCTTATGAATCAATTTATTAAT-3’) and Xyl-R (5’- GCGCTCGAGAAGATTGCCGTAAC-3’) (Sites of restriction enzyme HindIII and XhoI are underlined). The PCR products was purified, digested with HindIII and XhoI, and ligated into restricted plasmid pET22b(+). The recombinant vector was transformed into *E.coli* BL21(DE3) competent cells, and spread onto the LB-agar plate containing 50 μg ml^–1^ ampicillin. After incubation at 37 °C for overnight, positive colonies were verified by PCR, and the nucleotides were confirmed by sequencing. Before conducting xylanase expression, 2% of the overnight-incubated cultures were added into 50 ml LB medium with ampicillin and incubate at 37 °C until the OD_600nm_ reached about 0.5 ~ 0.6. Expression of xylanase was induced with 1.0 mM IPTG, followed by continuous incubation at 22 °C for 16 hrs. The supernatant was collected for activity detection by concentrating through the Vivaspin^®^ 20 centrifugal concentrator (GE Healthcare), and purified by a Ni-nitrilotriacetic acid (NTA) affinity chromatography column (His SpinTrapTM) (GE Healthcare, UK) based on the fused 6 × His tag.

### Characterization of recombinant GH11 xylanase from *E.coli*

The optimal pH for xylanase activity was determined by adding purified xylanase solution into different pH buffer system (from 4.0–10.0) and incubated at 50 °C for 10 min. The buffers used were citrate buffer (4.0–6.0), phosphate buffer (6.0–8.0) and glycine-NaOH buffer (8.0–10.0). The optimum temperature of the enzyme was determined by subjecting reaction mixtures into different temperatures ranging from 30 to 80 °C in a citrate buffer (pH 6.0) for 10 min. Substrate specificity of the purified xylanase was carried out by using different polymers, including birchwood xylan, beechwood xylan, pectin, carboxy methylcellulose (CMC), starch, under the optimal conditions. To determine the K_m_ and V_max_ for this recombinant xylanase, birchwood xylan in a concentration ranging from 1 to 5 mg ml^^−1^^ in 50 mM citrate buffer (pH 6.0) was set up to obtain the Lineweaver-Burk plot. The phylogenetic analysis of xylanases was performed by multiple alignment of xylanase protein sequences from different microbial species using ClustalX/MEGA softwares. The phylogenetic tree of xylanses was established with neighbor-joining method and bootstrapped 1000 times.

### Assay of xylanase activity

Xylanase activity assay was performed by adding 20 μl of enzyme solution (natural or recombinant enzyme) into 50 mM citrate buffer (pH 6.0) amended with 0.5% (w/v) of birchwood xylan at 50 °C for 10 min. The generated reducing sugar was measured by using the 3, 5-dinitrosalicylic acid (DNS) method^14^. One unit of xylanase activity was defined as the amount of enzyme that released 1 μmol of reducing sugar (as xylose equivalent) per min at above conditions. Concentration of proteins was measured by using the Lowry method with BSA as the standard.

### Nucleotide sequence accession number

The draft sequence data of *Cellvibrio mixtus* strain J3-8 are deposited at DDBJ/EMBL/GenBank databases under an accession number ALBT01000000. The version described in this paper is the first version. The full length of 16S rRNA gene of *C. mixtus* J3-8 and the xylanase-encoded gene are both deposited at GenBank with an accession number KC329916 and KC329917, respectively.

## Results

### Phylogenetic identification of *C. mixtus* J3-8 and optimization of its xylanase production

**Figure 1:**
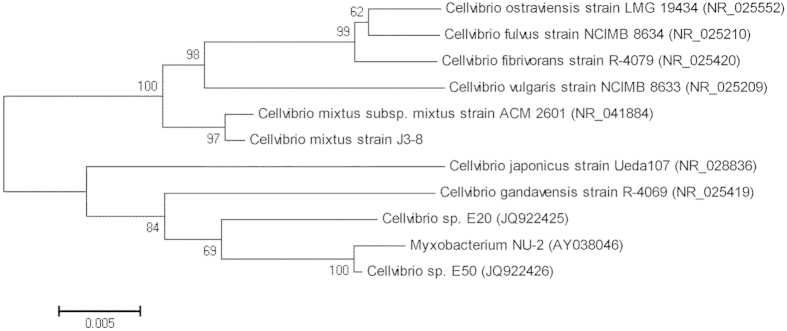
A neighbor-joining phylogenetic tree based on the 16S rRNA gene sequence of *Cellvibrio mixtus* strain J3-8 by using MEGA (Version 5.05). Distances determined according to Kimura’s two-parameter model and bootstrap values (>50%) based on 1,000 replicates are listed as percentages at nodes. Nucleotide sequence accession numbers are given in parentheses. Scale bar, 0.005 substitutions per 50 nucleotides.

With xylan as a substrate, a colony with relatively high xylanase activity was identified on an agar plate after Congo red staining. This colony was designated *C. mixtus* strain J3-8. The 16S rRNA gene sequence of strain J3-8 shows 99.5% identity (99.5%) to that of *C. mixtus* strain ACM 2601 (NCBI accession number AF448515), but <97% identity to that of other species in the same genus, such as *C. japonicus* and *C. vulgaris*. A phylogenetic tree based on the 16S rRNA gene sequences was established to show the relationship of the known *Cellvibrio* strains (Fig. 1).

**Figure 2:**
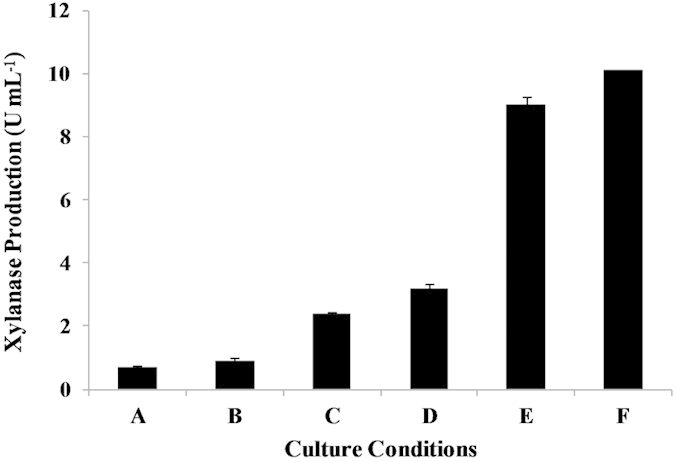
Optimization of xylanase production from *C. mixtus* J3-8. **A**: Original culture condition (25 °C, pH=7, 5 g L^−1^ xylan, without YE addition); **B**: Same condition as A, except changing temperature to 30 °C; **C**: Same condition as B, except changing pH to 8; **D**: Same condition as C, except adding 0.5% yeast extract; **E**: Same condition as D, except changing the xylan initial concentration to 10 g L^−1^; **F**: Same condition as E, except activating the inocula with 10 g L^−1^ of xylan.

**Figure 3:**
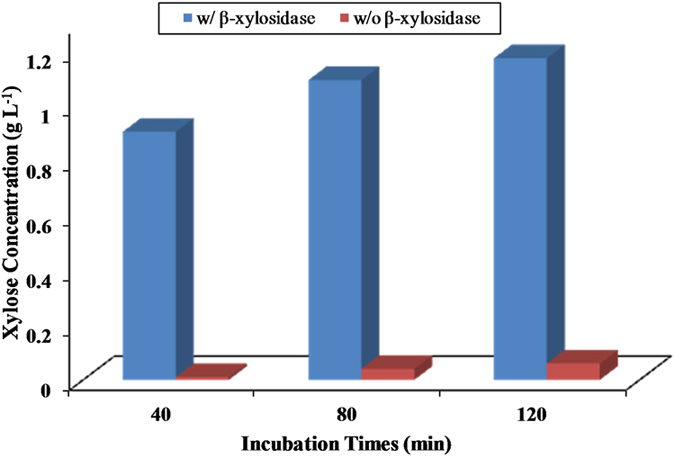
Xylan hydrolysis (3 g L^−1^) by crude extracellular enzymes (2 U ml^−1^ of xylanase activity) from strain J3-8. The concentration of generated xylose was detected with or without addition of commercial β-xylosidase (2 U ml^−1^).

To assess xylanase activity of culture *C. mixtus* J3-8, the extracellular enzyme was obtained from culture supernatant and its activity was detected to be only 0.68 U mL^−1^ after 5 days of incubation. After optimizing the culture conditions by stepwise examining different temperatures (25–35 °C), pHs (6–8), nitrogen sources (yeast extract, peptone or (NH_4_)_2_SO_4_), initial xylan concentrations (5–10 g L^−1^), and inoculum sizes (1%-10%), xylanase activity in the supernatant can be increased to 10.1 U mL^−1^ (Fig. 2) under optimal conditions (30 °C, pH 8.0, 10 g L^−1^ of initial xylan concentration, 10% of inocula, and with addition of yeast extract). This activity is comparable to previous reported microbial strains (e.g. *Jonesia* species, *Streptomyces* species, *Penicillium* speices) for natural xylanase production (Table 1). The result from strain J3-8 is consistent with previous studies^15^,^16^, showing that initial pH and initial concentration of xylan are important factors for improving extracellular xylanase production. Fontes *et al.* also reported that more xylanases, especially extracellular ones, can be produced by using xylan rather than glucose as a substrate^6^. On the other hand, extracellular enzymes from strain J3-8 were used for direct xylan hydrolysis (Fig. 3). Results showed that significant amount of xylose was produced only with the addition of extra commercial β-xylosidase, indicating that strain J3-8 could extracellularly produce xylanase and trace xylosidase.

### Genome sequencing and gene annotation

By using a high-throughput sequencing - whole-genome shotgun strategy, a total of 1,084,620 reads, counting up to 542.31 Mbp were received, providing 105-folds of coverage. The generated sequences were assembled into 152 contigs with an *N*_50_ length of 176,538 bps, and these contigs were assembled into 50 scaffolds. As a result, the draft genome of *C. mixtus* J3-8 consists of 5,171,890 bases, with a GC content of 46.66%, 32 tRNA genes, and 3 rRNAs (one 5S rRNAs, 16S rRNAs and 23S rRNAs). A total of 4,655 ORFs were obtained, which account to ~88.62% of total nucleotides. Among these genes, 2,845 protein-coding sequences (CDSs) (61.2% of the total) were annotated and identified by BLASTP search with the sequences from GenBank as the query. The identities of these genes are relatively low, of which 88.4% and 62.6% were below 90% and 80%, respectively. In addition, a total of 2,030, 1,771, 825 and 478 proteins were functionally annotated from UniProtKB/TrEMBL, KEGG, COG and UniProtKB/Swiss-Prot databases, respectively (Table S1). The comparison between strain J3-8 and the species from genus *Cellvibrio* with available genomic data is shown in Table 2.

### Construction of xylan metabolic pathway of *C. mixtus* strain J3-8

**Figure 4:**
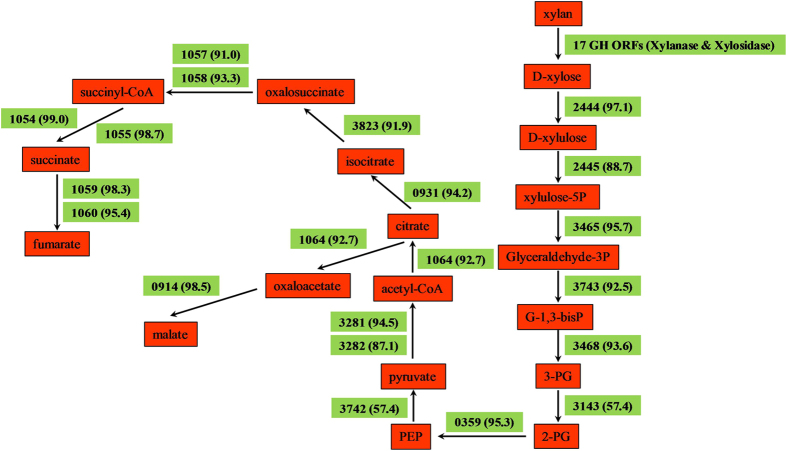
The metabolic pathway overview of *C. mixtus* J3-8 using xylan as substrate deduced from genome data. Abbreviations: G-1,3-bisP: Glycerate-1,3-bisphosphate; 3-PG: 3-phospho-glycerate; 2-PG: 2-phospho-glycerate; PEP: phosphoenolpyruvate. The numbers indicate the *C. mixtus* J3-8 annotation and highest identity (in the bracket) from GenBank based on the BLASTP, corresponding to the tag (CMXXXX) in Table S1.

A distinguished feature of the *Cellvibrio* genus is its capability to produce a series of hydrolytic enzymes for polysaccharides hydrolysis^6^,^8^,^17^. For the xylanase producing *C. mixtus* strain J3-8, the xylan metabolic pathway can be reconstructed from its genomic annotations (Fig. 4). Searching for genes related to xylan-hydrolytic enzymes in the genome of *C. mixtus* J3-8 led to the identification of 15 ORFs, which belong to four different glycoside hydrolase (GH) families based on the Carbohydrate-Active Enzymes (CAZy) database (Table 3). The most abundant GHs related to xylan hydrolysis are GH43 (8 ORFs) and GH11 (4 ORFs). However, these genes are only 41.3% to 88.8% identity to previous reported genes, including those from *Cellvibrio* species. In addition, five ORFs in the GH families are associated with known carbohydrate-binding modules (CBMs). Noteworthy, 9 out of the 15 ORFs are predicted to possess a signal peptide sequence for the extracellular protein secretion (Table 3).

In addition, ORFs coding for enzymes to utilize xylose were identified in strain J3-8. The pentose phosphate pathway (PPP) and the Enter-Doudoroff pathway (EDP) involved in xylose utilization could be deduced from the genome sequence (Fig. 4). Xylose is transformed into xylulose-5P via the gene cluster of xylose isomerase (EC 5.3.1.5, CM2444) and xylulokinase (EC 2.7.1.17, CM2445). The putative transketolase (EC 2.2.1.1, CM3465) functions as the transformation of xylulose-5P into Glyceraldehyde-3P (Angelov *et al.*, 2011), from which pyruvate is further formed through PPP. Citrate formation proceeds via pyruvate dehydrogenase components (CM3281-3282) to generate acetyl-CoA, which initiates the TCA cycle for central catabolic pathway (Fig. 4). Thus, *C. mixtus* possesses the complete pathway for the utilization of xylan and xylose.

### Analysis, cloning and expression of a GH11 xylanase from *C. mixtus* strain J3-8 in *E.coli*

**Figure 5:**
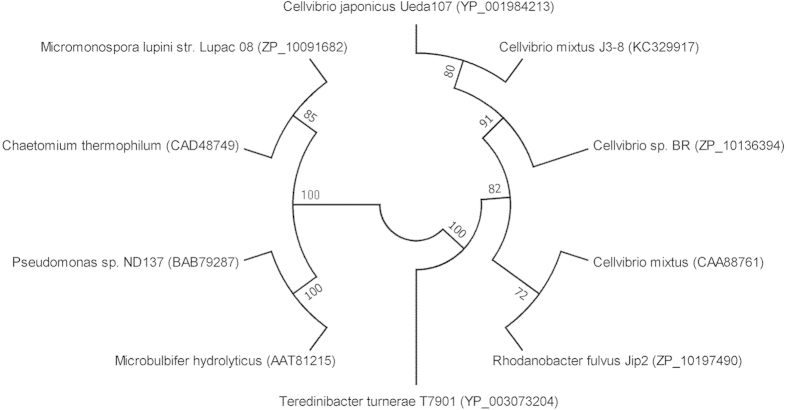
A neighbor-joining phylogenetic tree of xylanases based on the amino acid sequence by using MEGA (Version 5.05). Distances determined according to Kimura’s two-parameter model and bootstrap values (>50%) based on 1,000 replicates are listed as percentages at nodes. Nucleotide sequence accession numbers are given in parentheses.

**Figure 6:**
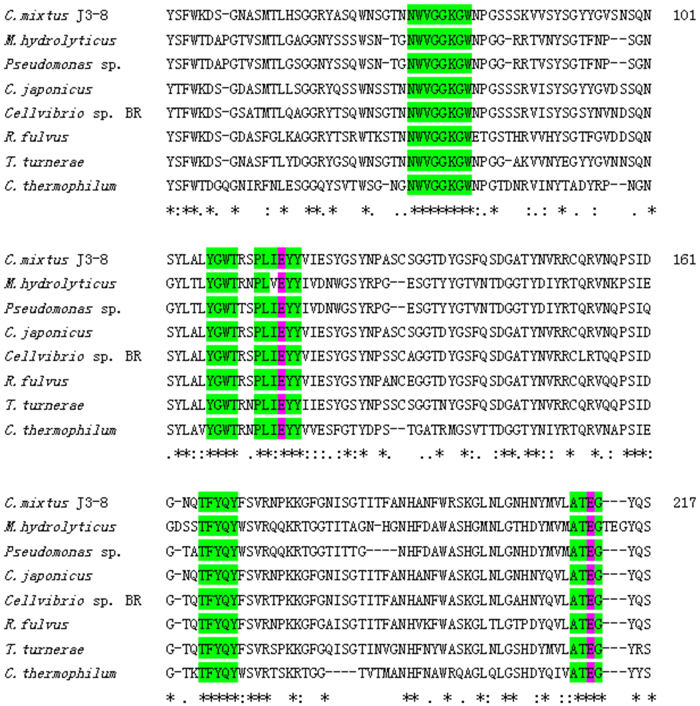
Sequence alignment of this novel family 11 xylanase. Highlighted blocks indicate the main conserved residues, and the pink color-highlighted amino acids (two glutamic acid residues) are predicted to be the catalytic site. The sequence number is based on *C. mixtus* J3-8 xylanase amino acid sequence.

As stated in previous section relatively, abundant amount of xylanases are found in the GH11 family in strain J3-8. Contrary to GH10 xylanases^18^, GH11 xylanases are the smallest xylanases, exhibiting several advantages, such as high substrate selectivity, high catalytic efficiency at various pHs and temperatures. Among the annotated four GH11 xylanase in strain J3-8 genome (Table 3), one xylanase-encoded gene (Tag No. CM1139, designated as Xyl_CM1139_) with smallest molecular weight and relatively high identity to reported xylanases was selected for cloning, expression and characterization in *E.coli*. The sequence homology of enzyme CM1139 showed only 82.7% identity at the amino acid level with its closest enzyme sequence of *C. japonicus* xylanase (YP_001984213) by using the ClustalW (Version 1.81) multiple sequence alignment program. It also shared 80.1% and 68.8% similarity with xylanase from *Cellvibrio* sp. strain BR (WP_007644724) and another *C. mixtus* (CAA88761). The phylogenetic tree was established with xylanase (Xyl_CM1139_) and other GH11 family members (Fig. 5). Five regions of amino acid residues (green color highlighted in Fig. 6) from these xylanases were found to be highly conserved, which are located in or surrounding the catalytic residues (two glutamic acids) (pink color highlighted in Fig. 6).

*E.coli* BL21 (DE3) cells harboring plasmid pET22b-Xyl_CM1139_ (encoding His-tagged Xyl_CM1139_ associated with a PelB signal peptide) were induced with 1 mM IPTG at 22 °C to express the complete ORF. After 16 hrs of incubation, the production of the recombinant extracellular Xyl_CM1139_ was detected to be 20.8 U ml^−1^. Purification of the Xyl_CM1139_ from crude medium was followed by two subsequent steps - ethanol precipitation and affinity chromatography. After purification, the specific activity of recombinant Xyl_CM1139_ was improved to be 48.0-fold (70.0 U mg^−1^) of the crude supernatants, together with a recovery rate of 19.2%. This purified enzyme revealed an apparent molecular mass of ~45 kDa, which is in good agreement with predicted molecular weight from its amino acid sequence (~38 kDa) fused with a PelB signal peptide (~7 kDa).

Characterization of the recombinant Xyl__CM1139__ was conducted in 50 mM citrate buffer (pH 6.0) at a temperature ranging from 30 to 90 °C. Besides citrate buffer, other buffers over a pH ranging from 4.0–10.0 were also tested. The optimal enzymatic activity of Xyl_CM1139_ was observed at the reaction conditions of 50 °C and pH 6.0, which are similar to those from most of the GH11 xylanases (Table 4). Results from the substrate specificity with other polysaccharides showed that both birchwood and beechwood xylan were the most suitable substrates for the recombinant xylanase (70.0 U mg^−1^). As predicted, this enzyme showed minute activities (1–2%) on CMC, starch and pectin. To further investigate the kinetics of the reaction catalyzed by Xyl_CM1139_ with birchwood xylan (1–5 mg ml^−1^) as the substrate, the *K*_*m*_ and *V*_max_ estimated by a Lineweaver-Burke plot were determined to be 6.0 mg ml^−1^ and 6.3 U mg^−1^, respectively. Table 4 shows the comparison between Xyl_CM1139_ and those reported recombinant GH11 xylanases from other microbial strains.

## Discussions

A novel aerobic xylanase-producing bacterium *Cellvibrio mixtus* strain J3-8 was isolated and characterized in this study, which is capable of naturally producing xylanase (10.1 U ml^−1^) – comparable to that of previous known bacteria or fungi. Genomic sequence analysis identified 2,845 annotated ORFs, exhibiting relatively low similarity (83.8% of ORFs <90% of similarity) with previously reported xylanolytic genes in other *Cellvibrio* species. In addition, the genomic size of *C. mixtus* strain J3-8 is much larger than the other three *Cellvibrio* species (Table 2), resulting in the relatively lower annotation percentage from those detectable ORFs. This result also suggests that strain J3-8 is highly different from other reported *Cellvibrio* species by possessing abundant novel genes in those non-annotated fragments. Furthermore, a large amount of GHs encoded genes were found in the genome of *C. mixtus* J3-8, and the relatively low identity of these enzymes with known ones indicates their novelty.

A few hydrolytic genes from *Cellvibrio mixtus* have been described 6,8,9,11,17, however, only limited information is available, especially on xylanolytic enzymes either natural or recombinant ones. Analysis on the xylanolytic GHs from the genome indicates that the expression of xylan degradation-related genes in *C. mixtus* J3-8 is not accomplished on a basis of the gene cluster or a cellulosomal enzyme system (Table S1). This is in accordance with the observation from strain *C. japonicus* that its enzymes do not assemble into large multienzyme cellulosome-like complex and fully secrete into extracellular environment separately^2^. With 15 putative ORFs encoding xylanases or xylosidases in the genome of *C. mixtus* J3-8, the signal peptide (SP) structure of these enzymes demonstrates that more xylanase-encoded genes were detected with SP rather than xylosidase-encoded ones. This observation explains results from Fig. 3 that only few amount of β-xylosidase was present in crude extracellular enzymes received from *C. mixtus* J3-8, even though the number of β-xylosidase-encoded genes are much higher in the whole genome. In addition, as CBMs are usually considered to enhance the efficiency of hydrolytic enzymes by mediating prolonged and intimate contact between the respective catalytic module and its target substrate^19^, more CBMs in GHs’ ORFs from strain J3-8 were observed in xylanase-encoded ORF rather than in xylosidase from the whole genome. It is reasonable that xylanases require the CBMs to bind to internal structure of xylan for enhancing their hydrolysis efficiency. Three types of CBMs (CBM2, 10 and 15) structures were detected from the binding domain of identified xylanases in strain J3-8. Among them, CBM2 is the largest prokaryotic CBM family, which contains CBM2a (for binding cellulose) and CBM2b (for binding xylan), and CBM2b was reported to match the structure of the binding site to the helical secondary structure of xylan through its specific ligands for protein-carbohydrate interaction^20^. CBM10 is found as the cellulose-binding module usually appended to xylanase to facilitate their contact for hydrolysis of cellulosic materials^21^,^22^; however, it seems not functional in strain J3-8 due to absence of cellulose activity. CBM15 is considered as a specific module that only present in *Cellvibrio* genus, such as *C. japonicus*, *C. mixtus* and *Cellvibrio* sp. BR^23^,^24^, and its major role is to bind the xylan by particularly well adapting to xylanase to highly exposed regions of xylan^19^.

In the genome of *Cellvibio* species, several GHs are identified to contain xylanases, including GH10 and GH11. GH10 seems to be easily detected in bacteria rather than GH11 xylanases. Compared to fungi, the amount of currently characterized GH11 (425 cases) in bacteria is much lower than that of GH10 xylanases (938 cases) from CAZy database (http://www.cazy.org). However, GH11 was discovered to be more abundant in strain J3-8 than GH10 xylanases, and the xylanase in GH11 is considerably more active than GH10 xylanase due to its high substrate specificity, great stability and plasticity, especially the small sizes^18^,^19^. The heterogeneously and functionally expression of GH11 xylanase in *E.coli* shows similar properties as GH11 xylanases from other species, suggesting the main characteristics of GH11 xylanase: low molecular weight, alkaline p*I* value, and slightly acidic optimum pH. As described in Paes *et al.*^18^, GH11 xylanases display a jelly-roll super-fold structure with highly conserved domains, and Xyl_CM1139_ from *Cellvibrio mixtus* J3-8 was observed with five conserved domains^18^. The enzyme active site of Xyl_CM1139_ involving glutamic acid (E116, function as proton donor) and the other glutamic acid (E213, function as nucleophile) are located in the third and fifth domain, participating in a typical catalysis of GH11 family (Fig. 6)^25^. Comparing with other known GH11 xylanases on their main structural characteristics^18^, it is highly possible that the characterized xylanase from strain J3-8 shows two long loops between two β-sheets in its secondary structure of this xylanase, similar to that from a xylanase in a fungal species *Neocallimastix patriciarum*^26^, and such kind of xylanase structure is seldom reported in bacterial species.

From the above analysis, *C. mixtus* strain J3-8 shows distinctive difference from known species, which may be due to limited studies on bacterial strains present in snails. Snails could be an excellent host for bacteria possessing hydrolytic enzymes because snails usually feed on edible plant matters, including fruits, vegetables, grass, leaves as well as decaying organic materials^27^, and the nature of these food requires an effective cellulase/xylanase system for hydrolysis and digestion^27^,^28^,^29^. Thus isolating microorganisms capable of producing hydrolytic enzymes from snail is highly likely. However, studies on bacterial strain in snails are limited^27^,^28^, especially on genomic analysis and functional gene identification. The discovery of novel strain J3-8 from a snail not only provides an approach to investigate new microbes from those phytophagous organisms (e.g., insects), but also provides genomic information regarding to valuable xylanases, which shows potential in exploring strain J3-8’s other novel hydrolytic genes for biotechnological and industrial applications. Meanwhile, the genome should be equally valuable in revealing the relationship between hydrolytic enzymes and CBMs, exhibiting benefit for improving the efficiency of polysaccharides hydrolysis.

## Author Contributions

Wu, Y.R. and He, J. designed the experiments; Wu, Y.R. conducted the experiments and prepared all the figures and tables; all authors contributed to the manuscript writing and reviewed the manuscript.

## Additional Information

**How to cite this article**: Wu, Y.-R. & He, J. Characterization of a xylanase-producing *Cellvibrio mixtus* strain J3-8 and its genome analysis. *Sci. Rep.* **5**, 10521; doi: 10.1038/srep10521 (2015).

## Supplementary Material

Supplementary Table S1

## Figures and Tables

**Table 1:** Comparison of xylanase production by known microorganisms

**Species**	**Xylanconcentration**	**Cultivationconditions**	**Xylanaseactivity (U ml**^−**1**^)	**References**
*Bacillus coagulans*	10 g L^−1^	Shake flask,45 °C, pH 7.0,1 day	24.2	10
*Gracilibacillus* sp. TSCPVG	7.5 g L^−1^	Shake flask,30 °C, pH 7.5,4.5 days	9.6	15
*Jonesia denitrificans* BN13	7 g L^−1^	Shake flask,37 °C, pH 7.0,2 days	2.5	16
*Streptomyces* sp. strain AMT-3	10 g L^−1^	Shake flask,30 °C, pH 7.0,10 days	10.3	25
*Streptomyces malaysiensis*	10 g L^−1^	Shake flask,30 °C, pH 7.0,6 days	11.9	25
*Penicillium kloeckeri* NRRL1017	10 g L^−1^	Shake flask,30 °C, 5 days	12.2	5
*Cellvibrio mixtus* J3-8	10 g L^−1^	Shake flask,30 °C, pH 8.0,5 days	10.1	This study

**Table 2:** Comparison of genomic data from different *Cellvibrio* species.

	*C. mixtus* J3-8*	***C. japonicus***	***C. gilvus***	*Cellvibrio* sp. BR*
**Genome Size (Mbp)**	5.17	4.58	3.53	4.85
**GC (%)**	46.7	52.0	73.8	48.8
**Total ORFs**	4,655	3,812	3,263	4,349
**Annotated ORFs**	2,845	3,750	3,164	4,304
**Annotation Percentage (%)**	61.1	98.4	97.0	99.0
Number of GH ORFs				
GH10	2	4	6	2
GH11	4	2	2	0
GH 30	1	2	2	0
GH43	8	14	7	9

^*^Draft genomic sequence.

**Table 3:** Identification of glycoside hydrolases (GHs) ORFs involved in xylan hydrolysis in the genome of *C. mixtus* J3-8.

**ORF ID#**	**Known activities for GH domain by BLASTP**	**Best identity**	**GH family**	**CBMs**	**Signal Peptide**
CM1128	Xylanase	67.9	GH10		−
CM1139	Xylanase	82.7	GH11		+
CM1145	Xylanase	75.8	GH11	CBM10	+
CM2255	Xylanase	72.9	GH30	CBM2	+
CM2451	Xylanase	72.9	GH10	CBM15	−
CM3064	Xylanase	81.1	GH11		+
CM3067	Xylanase	77.2	GH11	CBM10	+
CM1137	Xylosidase	61.5	GH43	CBM2	+
CM2382	Xylosidase	55.8	GH43		−
CM2394	Xylosidase	70.8	GH43		−
CM2398	Xylosidase	57.3	GH43		−
CM2407	Xylosidase	88.8	GH43		+
CM2452	Xylosidase	61.0	GH43		+
CM2455	Xylosidase	75.4	GH43		−
CM3057	Xylosidase	73.5	GH43		+

**Table 4:** Main characteristics of reported recombinant GH11 xylanases from different bacteria.

**Microorganisms**	**MW (Da)**	**p**I*	**pH**_**opt**_	**Temp**_**opt**_**(°C)**	***K***_***m***_ **(mg ml**^−**1**^)	**Reference**
***Bacillus licheniformis*** **I5**	23,401	9.96	7.0	50	—	30
***Bacillus subtilis*** **str. R5**	23,359	9.51	6.0	40-50	4.5	31
***Cellulomonas flavigena*** **CDBB-531**	35,110	9.61	6.5	55	0.98	32
***Clostridium cellulovorans***	54,000	7.92	5.0	60	—	25
***Nonomuraea flexuosa***	23,800	—	6.0	80	6.0	33
***Ruminococcus albus***	74,600 (w/CBM)	4.51	6.5	50	—	34
***Streptomyces*** **sp. S27**	25,202	9.48	6.5	65	2.3	35
***Thermobifida halotolerans***	34,112	9.34	9.0	70	3.5	36
***Cellvibrio japonicus***	37,912 (w/CBM)	8.49	—	—	—	2,37
***Cellvibrio mixtus***	66,405 (w/CBM)	6.51	—	—	—	23
***Cellvibrio mixtus*** **J3-8**	38,233	8.65	6.0	50	6.0	This study

^*^p*I*: isoelectric point
